# Patient’s willingness to pay for improved community health insurance in Tanzania

**DOI:** 10.1016/j.hpopen.2024.100130

**Published:** 2024-09-29

**Authors:** Kassimu Tani, Brianna Osetinsky, Sally Mtenga, Günther Fink, Fabrizio Tediosi

**Affiliations:** a Ifakara Health Institute, Dar es Salaam, Tanzania; b Swiss Tropical and Public Health Institute, Allschwil, Switzerland; c University of Basel, Basel, Switzerland

**Keywords:** Community health insurance, Willingness to pay, Contingent valuation method, Medication, Tanzania

## Abstract

Globally, achieving universal health coverage remains significant challenge. Health insurance coverage in low- and middle-income countries is still low with only a few African countries managed to reach 50% coverage. This study aimed to investigate the factors influencing patients’ willingness to pay (WTP) for medication and various versions of the improved Community Health Insurance Fund (iCHF) in Tanzania. A facility-based cross-sectional study was conducted in all hospitals, health centres, and eight randomly sampled dispensaries, sampling participant from the queue, one out of every three patient based on their order of entry into consultation room, and interviewed 1,748 patients in Kilombero and Same districts in Tanzania. We used multi-stage Contingent Valuation Methods exploring data collected during client exit interviews. We employed a random utility model and estimated WTP through an ordered logit model. The independent variables were; patient’s gender, age, marital status, education, employment status, Non-Communicable Disease (NCD) status, health insurance status, and the type of healthcare facility level. Our findings revealed that most patients exhibited a WTP of an amount equivalent to the current iCHF premiums and would also be willing to pay for an augmented iCHF premium inclusive of additional medication coverage. Upon adjusting for demographic characteristics, we observed that patients enrolled in an insurance program or benefiting from user fee waivers demonstrated a lower WTP for medication, while those with non-communicable diseases (NCDs) and seeking care in private facilities exhibited a higher WTP. Furthermore, patients with a secondary education level or above generally displayed higher WTP for premiums. Conversely, patients enrolled in private insurance and availing user fee waivers, along with those accessing care in public facilities, demonstrated a lowered WTP for iCHF premiums. These results highlight the need for targeted interventions to address systemic deficiencies and improve access to medicines. Our conclusions is that policies considering NCD status, education levels and income status are important when designing health insurance schemes for the informal sector in Tanzania, with the goal of increasing uptake of CHF.

## 1 Introduction

Globally, achieving universal health coverage (UHC) remains a significant challenge [1], [2], [3], [4], particularly in low- and middle-income countries (LMICs). Mobilizing public revenues for financing health systems in these countries is difficult. Expanding health insurance coverage is one strategy that can assist LMICs in progressing towards UHC, although it is not sufficient on its own, especially where health insurance coverage remains low [5], [6], [7], [8], [9], [10]. In Africa, only a few countries have managed to enroll more than 50% of the population after institutionalized mandatory health insurance for all [11], [12], [13]. To expand insurance coverage, countries have implemented a variety of strategies, balancing needed health care benefits against feasible revenue collection [9], [14], [15]. One way to increase health insurance coverage is for the countries to strengthen insurance policies and schemes acceptable and designed to cover informal sectors and rural population [16], [17], [18], [19]. These schemes primarily are government’s non-profit scheme targeting identified under-insured populations.

Tanzania has a compulsory National Health Insurance Fund (NHIF) scheme for civil servants, which has been extended to everyone on voluntary basis [20], and a voluntary community health insurance scheme called “improved Community Health Funds” (iCHFs). The annual iCHF premium is cheaper than the NHIF one aiming to reach the vulnerable rural population [20], [21], [22], [23]. The iCHF aims to cover the cost of essential health services, including laboratory tests and prescribed drugs available at public facilities [24]. However, the percentage of population enrolled and covered by health insurance in informal sectors remains low despite government efforts to promote the schemes [6], [16]. The iCHF covers around 25% of the population in Tanzania. In total, 32% of the Tanzanian population possesses health insurance [14], [16], [25]. In Same and Kilombero districts iCHF coverage was 7.5% in 2021 and 20% of the population had any type of health insurance coverage [26].

Expanding enrolment in voluntary health insurance remains challenging due to low new recruitment and fluctuating dropout rates [16], [17], [23]. Previous studies have also highlighted poor management, lack of confidence in schemes' design and inadequate benefit packages as reasons for low insurance enrollment [5], [7], [14], [18], [27], [28]. These challenges are compounded by the perceptions of low quality of services, frequent medicines stock-outs [6], [7], [26], [29], [30], [31], [32], and a lack of comprehensive information about the benefits of health insurance [17], [33]. Additionally, on the demand side, the population may be reluctant to enroll/re-enroll in the iCHF due to significant health system shortcomings, which remain major obstacles to the utilization of health services, as highlighted by several studies [5], [27], [29], [32]. This is particularly challenging for people with hypertensions, diabetes mellitus, chronic kidney diseases, cancers and other chronic conditions, who require frequent care and are at greater risks of catastrophic consequences due to limited access to health services [34], [35], [36], [37].

In pursuit of UHC, the Tanzanian government has undertaken significant health insurance policy reforms. On November 2nd, 2023, the Parliament passed a bill proposing a mandatory national scheme to cover the extensive and diverse informal sector, which became law on December 5, 2023 [38]. The next step in implementing mandatory health insurance for all involves setting premiums and benefit packages. To achieve this national goal, it is of interest to investigate how much patients are willing, able and prefer to pay for health insurance premiums.

In the previous iteration of the countrywide community health insurance program, the uniform annual premium for the iCHF was set at Tsh 30,000 ($13), per household covering a maximum of six members. An exception was made for Dar es Salaam, where the premium was Tzs 150,000 ($65) [23]. Throughout the pilot phase and scaling up of the voluntary scheme, numerous studies were conducted to evaluate the redesigning of the scheme [5], [6], gauge the WTP for the premiums and identify recruitment and uptake challenges [7], [28]. While analyzing decision regarding enrollment, previous studies predominantly focused on binary dependent variables at the household level, neglecting to explore individual patient perspectives at the point of care, including preferences for accessing supplies beyond public health facility pharmacy. This study analyze the variation in WTP among a sample of health services users [39], [40], [41]. The primary aim is to identify the factors influencing patients' WTP for medication and premium versions of the iCHF in Tanzania. This approach provides valuable insights for policymakers and stakeholders involved in designing premiums and benefit packages for inclusive health insurance.

## 2 Theoretical underpinning of willingness to pay

This study utilizes consumer choice and welfare economics theories, which provide a framework for understanding consumers WTP. According to the consumer choice model, consumers make decisions by considering their budget constraints and the trade-off needed to maximize their utility. The welfare economics theory highlights the benefit an individual gains from using a service or intervention, and can be defined by their maximum WTP for that service or intervention [42], [43]. When illustrating individual choices and WTP for improved services, factors such as ability to pay, potential benefits, education level, and health status are important considerations [44], [45], [46], [47]. Assuming that there are further improvements to existing community health insurance that expands the benefits coverage, the maximum WTP represents the highest amount an individual would pay to enroll in the insurance to gain full benefit package while maintaining the same overall level of wellbeing. If an individual had to pay more than this maximum value, the loss of income would outweigh any gain in well-being. To estimate an individual's WTP, one starts with a minimum amount and a given state of well-being benefits. As the amount increases, the well-being benefits also increase until they reach a maximum, beyond which additional benefits do not further increase the WTP (S1 Figure). In this context, an individual’s WTP reflects the value they assign to the improved community health insurance benefit packages. This value varies between individuals and may depend on demographic and socioeconomic factors and individual health status (S2 Figure).

## 3 Methods

### 3.1 Study design and setting

The government of Tanzania expanded enrollments and insurance coverage. Further efforts are needed to explore patients' WTP for community health insurance schemes that cover a wider population. This study used client-exit interview survey data, collected between September 2020 and January 2021 in two rural districts of Tanzania: Same district in the northern region and Kilombero district in the south-eastern part of the country (S3 Figure). Both districts are characterised by subsistence farming, livestock rearing, and fishing as the primary livelihoods of the population. These districts have been among the first to implement community health fund in 2001, aiming to improve social health protection and access to health care [17], [23]. Additionally each district has a private hospital serving as the main referral center for health services.

### 3.2 Sampling

The survey was administered at all public and private tertiary and district hospitals (n = 4), all health centres (n = 16), and a random sample of eight dispensaries in both Kilombero and Same districts (n = 16). To ensure a representative sample of dispensaries, we randomly selected one dispensary from each ward containing a health centre. All patients aged 18 and above who were entering the outpatient clinic on the day of the survey were eligible for recruitment, regardless of type health services they were seeking. We employed operationally random sampling by selecting participants from the queue, recruiting one out of every three patients based on their order of entry into the consultation room, ensuring each had an equal probability of selection. This method is more efficient and simpler to implement than other random sampling approaches. Additionally, it minimizes the bias associated with consultation length that can occur when sampling patients at the end of consultation [48]. The sample size was calculated using a modified Cochran approach based on the hypertension and diabetes prevalence for rural Tanzanian adults and population estimates for the two districts. The full detail of the survey and sample size have been described in previous work [39], [40]. Clients were interviewed after receiving all services and collecting prescribed medications at the facility pharmacy if they were available. Across both districts, the total sample size resulted in 1748 respondents.

### 3.3 Willingness to pay assessment

We examined patients' WTP for a health insurance such as the iCHF, that would also cover refilling chronic care medications on a monthly basis. We assessed WTP by asking participants how much, in addition to iCHF premium, they would be willing to pay out-of-pocket each month for their medications if their doctor prescribed them. By utilizing the contingent valuation method (CVM), we elicited the maximum iCHF premium and out-of-pocket amount participants would be willing to pay each month for their prescribed medications. The CVM is a widely employed technique to obtain information on individuals' maximum WTP for specific goods or services, utilizing hypothetical scenarios. To select the bids, we conducted a thorough review of the iCHF as currently designed, as well as relevant literature. We began with a bid of 5,000 Tsh (2.2 USD) and adjusted the bid up or down based on the participant's response until we reached 10,000 Tsh (4.4 USD) maximum or 1000 Tshs (0.4 USD) minimum bid designed based on pilot responses. In cases where the respondent declined the stated bid, we decreased the bid until the participant agreed or until we reached the minimum bid in the study design.

To determine the importance of access to private pharmacies in mitigating stock-outs, participants were asked about their WTP for an improved iteration of the iCHF that included this feature. This research was guided by existing literature highlighting the challenges of poor service delivery and frequent stock-outs of drugs and medical supplies in public health facilities, which have been identified as significant barriers to iCHF enrolment [7], [49]. The study started with an initial bid of 60,000 Tanzanian shillings (equivalent to USD 25.9), which was subsequently adjusted either upwards or downwards. Participants who accepted the initial bid were presented with progressively higher bids up to a maximum bid of 120,000 Tanzanian shillings (USD 51.9). Conversely, respondents who rejected the initial bid were presented with the minimum bid, which was set at 30,000 Tanzanian shillings (USD 13.0). The survey ended with an open-ended question asking all participants to state the maximum amount they would be willing to offer (S1 Table).

We chose this method of presenting bids with “Yes” and “No” responses to better accommodate the general literacy level of the rural population [50]. Majority of this demographic group residing in rural area often experiences poor services resulted from frequent stock-outs of drugs and medical supplies at health facilities sought care [51] making it crucial to use a straightforward and easily understandable survey format. Additionally, the simplicity of YES/NO questions helps ensure accurate responses and facilitates easier analysis, enabling us to effectively assess the community's WTP for improved health insurance options.

### 3.4 Data collection and management

Data was collected by experienced enumerators on quantitative research. Prior to data collection, the enumerators received comprehensive training on the study objectives, research methods, including the CVM, and were reminded of human research ethics. The data collection tools were pilot-tested, and necessary adjustments on bids maximum and minimum level were made based on feedback received. The pilot demonstrated that respondents understood the bid choices clearly and could express their preferences effectively.

The data was captured using electronic devices (tablets) programmed with ODK software. All responses were directly entered into the tablets and transferred to the secure Ifakara Health Institute data server. To ensure data quality, qualified research staff frequently reviewed the data for consistency, and immediate feedback was provided to enumerators.

The data collection tool captured information on respondents' demographic and socio-economic characteristics, enrollment in social health protection schemes, health-seeking behavior, and health status before proceeding to the WTP. The WTP preferences section followed an introduction and hypothetical scenarios.

### 3.5 Client-exit variables

In examining patients’ WTP, the outcome variables for this study were WTP for medication and augmented iCHF premiums. Participants were categorized based on their WTP for various monthly medication expenditures and augmented iCHF with medication package scenarios. WTP for monthly medication for chronic conditions was stratified into predefined categories: <1000; 1000 − <2500; 2500 − <5000; 5000 − <10000; and > 10000 Tanzanian shillings. Similarly, iCHF premiums were categorized into: <30000; 30,000 − <60000; 60,000 − <90000; 90,000 − <120000; and > 120000 Tanzanian shillings (S2 Table).

The independent variables included gender, age, marital status, education, employment status, NCD status, health insurance status, and the type of health facility where the respondent sought care (S3 Table).

### 3.6 Data analysis

We present descriptive statistics detailing the demographic and socioeconomic profiles as well as WTP for OOP expenses, the current iCHF scheme or an augmented iCHF of the participants, alongside their WTP for hypothetical healthcare scenarios. To investigate the relationships between these characteristics and WTP, we conducted a chi-squared test of homogeneity. Participants enrolled in the NHIF scheme were omitted, as their package was more comprehensive and included the option of obtaining medicine at NHIF accredited private pharmacy, if their prescribed medicine was unavailable [14], [26].

Given that the dependent variable (WTP) consisted of ordered values and exceeded two categories, we employed ordered logistic regression. This model was chosen as it accounts for the ordinal nature of the WTP categories, assumes that the relationship between each pair of outcome groups is statistically similar, and provide more efficient estimates and clear interpretations [44], [52]. The proportional odds assumption was tested and confirmed using the Brant test (p-value = 0.16) (S4 Table).

The regression models examined the categorized WTP for medication and iCHF premiums in relation to the patient’s gender, age, marital status, education, employment status, NCD status, health insurance status, and the type of health facility respondent sought care.

The regression model specification is show in equation (1)

$$Logit\left(P(WTP\leq j)\right)=\alpha j+\beta 1gender+\beta 2agecategory+\cdots \beta ktypeofhealthfacility$$

where:

• $\left(P(WTP\leq j)\right)$ is the cumulative probability of the dependent variable WTP being in category j or lower for medication or iCHF premiums.
• αj is the threshold for category j.
• β1, β2, …, βk are the coefficients for the independent variables gender, age, ……, type of health facility.

The selection of independent variables was guided by economic theory and existing literature in the field, ensuring the inclusion of variables theoretically and empirically linked to the research question [32], [36]. We employed the backward elimination method to determine the optimal model specification, systematically removing non-significant variables until a parsimonious model was achieved [53], [54]. This approach aids in reducing multicollinearity and enhances the interpretability and robustness of the model.

To evaluate the adequacy of our model specification, we employed the “*linktest*” command in Stata. This test assesses the functional form and specification of the model by examining the relationship between predicted probabilities and the observed outcomes. By doing so it ensure that, the model accurately captures the relationship between the independent variables and the outcome variable. The results of these linktests are presented in Supplementary Tables (S5 and S6 Tables).

Recognizing that our observations were nested within hospitals, health centres, and dispensaries, we employed clustering in our analysis. Clustering adjusts for potential within-group correlation, resulting in more accurate standard errors and inferential statistics. Empirically, intraclass correlation coefficient were 0.02 for medication and 0.08 for the augment iCHF model.

Adjusted odds ratios (AORs) and corresponding 95 % confidence intervals were computed to estimate the associations between independent variables and the outcome of interest. These measures provided a quantitative assessment of the magnitude and direction of the relationships under investigation.

## 4 Results

### 4.1 Descriptive statistics

A total of 1,748 patients participated in the survey. Among them, 39 % possessed health insurance, with 12 % enrolled in the iCHF. Generally, patients expressed willing to pay out-of-pocket for medications (66 %), the current iCHF program (73 %), and the current iCHF premium combined with an additional drug package (77 %). Notably, 77.2 % and 80.5 % of patients not currently enrolled in any health insurance scheme expressed a WTP for the current iCHF premium and its extension, respectively (Table 1).

**Table 1:** Socio-economic and demographic composition of the respondents.

**Variables**	**Willing to pay for Medication out of pocket**		**Willing to pay for Amount equivalent to Current iCHF**		**Willing to pay for Current iCHF and additional medication**	
	**n = 966 (66 %)**	**P-value**	**n = 956 (73 %)**	**P-value**	**n = 1291 (77 %)**	**P-value**^1^
**Age (Years)**						
Below 35	358 (54.3)	0.69	430 (76.7)	<0.01	515 (82.5)	<0.01
35–––55	319 (56.4)		362 (78.0)		454 (84.2)	
Above 55	289 (56.5)		164 (57.8)		322 (74.9)	
**Gender**						
Female	628 (56.8)	0.19	613 (73.2)	0.82	829 (81.3)	0.67
Male	338 (53.5)		343 (72.7)		462 (80.5)	
**Education**						
No education	173 (58.6)	0.38	147 (61.5)	<0.01	197 (70.9)	<0.01
Primary completed	550 (55.7)		604 (75.6)		750 (81.6)	
Secondary and above	243 (53.5)		205 (75.6)		344 (86.9)	
**Marital status**						
Never married	131 (48.9)	<0.01	144 (64.0)	<0.01	181 (72.7)	<0.01
Married	641 (54.7)		686 (77.8)		821 (84.7)	
Separated	194 (65.3)		126 (62.1)		189 (73.8)	
**Locality**						
Urban	763 (60.8)	<0.01	697 (76.2)	<0.01	971 (83.7)	<0.01
Rural	203 (42.1)		259 (65.7)		320 (73.9)	
**Occupation**						
Employed	200 (48.1)	<0.01	200 (72.7)	0.04	299 (81.7)	0.02
Farmer	539 (57.7)		547 (72.3)		689 (78.6)	
Self-employed	177 (57.8)		197 (77.6)		254 (87.0)	
Retired	50 (61.7)		12 (52.2)		49 (83.1)	
**Health condition reported**						
Not NCDs	630 (52.2)	<0.01	745 (74.6)	0.02	919 (81.1)	0.91
NCDs	336 (63.5)		211 (68.1)		372 (80.9)	
**Social protection**						
OOP	539 (58.6)	<0.01	713 (77.2)	<0.01	744 (80.5)	<0.01
iCHF	122 (57.3)		167 (77.3)		179 (82.9)	
NHIF	234 (53.7)		−		283(100.0)	
Private insurance	13 (37.1)		5 (14.3)		8 (22.9)	
Exemption	58 (43.3)		71 (53.0)		77 (57.0)	
**Districts**						
Kilomero	662 (76.5)	<0.01	531 (77.3)	<0.01	688 (82.5)	0.12
Same	304 (34.9)		425 (68.3)		603 (79.4)	
**Facility ownership**						
Private	408 (62.6)	<0.01	335 (84.2)	<0.01	536 (91.0)	<0.01
Public	558 (51.7)		621 (68.2)		755 (75.2)	
**Facility level**						
Dispensary	88 (53.3)	0.49	122 (80.8)	<0.01	145 (89.0)	<0.01
Health centre	643 (55.1)		638 (69.4)		824 (77.2)	
Hospital	235 (58.1)		196 (82.0)		322 (88.9)	
**Age Years (mean) (SD)**	44.6 (17)	−	40.1 (15)	−	42.8 (16)	−

1 P-value was calculated by using the chi-square test.

**Fig. 1:**
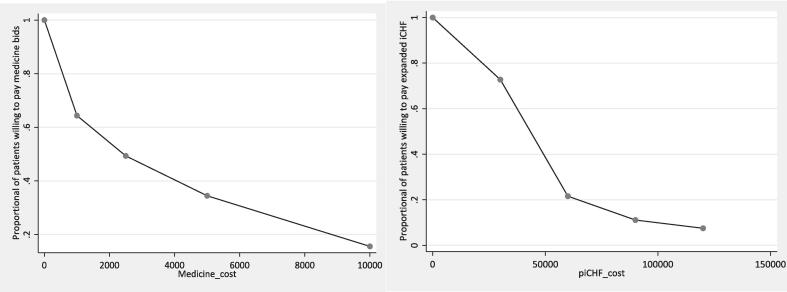
Willingness to pay as per elicited bids for Medicines and iCHF premium with medicines package to the level of private pharmacy.

**Fig. 2:**
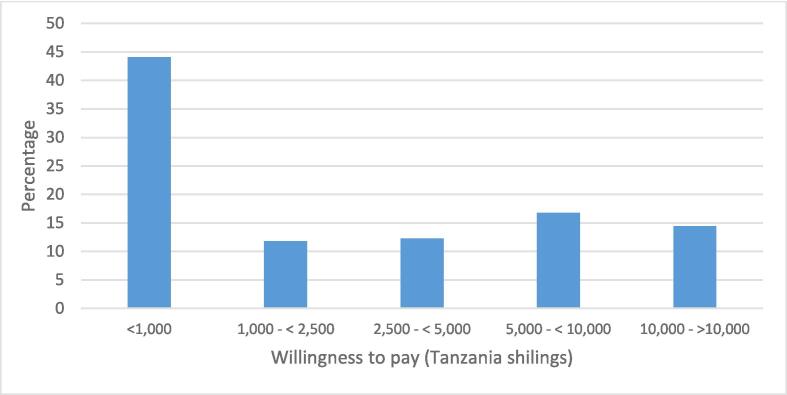
Distribution of willingness to pay for medicines.

In this study, a total of 1,310 patients were asked about their WTP for drugs. Of these, 855 patients (65.3 %) responded positively to the initial offer of 1000 Tshs, while 455 (34.7 %) responded negatively. Of the 855 patients who accepted the first offer, 643 (75.1 %) responded positively to the second offer of Tshs 2,500, while 212 (24.9 %) responded negatively. Of these 643 patients, 433 (67.4 %) agreed to the third bid level of 5,000 Tshs, while only 188 (29.2 %) responded positively to the highest bid level of 10,000 Tshs (S4 Figure). Regarding payment for the iCHF premiums with an additional medication package outside of public health facility pharmacies, a total of 1,307 patients were surveyed. Among them, 989 patients (75.6 %) responded positively to the lowest bid of 30,000 Tshs, which is equivalent to the current premium, while 318 (24.4 %) responded negatively. Among those who responded positively to the lowest bid, 265 patients (26.8 %) agreed to the second bid level of 60,000 Tshs. Furthermore, 124 patients (12.5 %) responded positively to the third bid level of 90,000 Tshs, and 79 patients (8.0 %) responded positively to the highest bid level of 120,000 Tshs (S5 Figure). Fig. 1 provides a visual representation of the relationship between medication and iCHF bid acceptance rates and their corresponding prices. It reveals a typical demand curve, indicating an inverse relationship between the bid price and acceptance rate. Specifically, as the bid price increased, the number of patients accepting the bid decreased. In Fig. 2, it can be observed that the majority of respondents who expressed positive bids for medication were willing to pay for the highest bids, which accounted for 16.8 % and 14.4 % of the respondents.

**Fig. 3:**
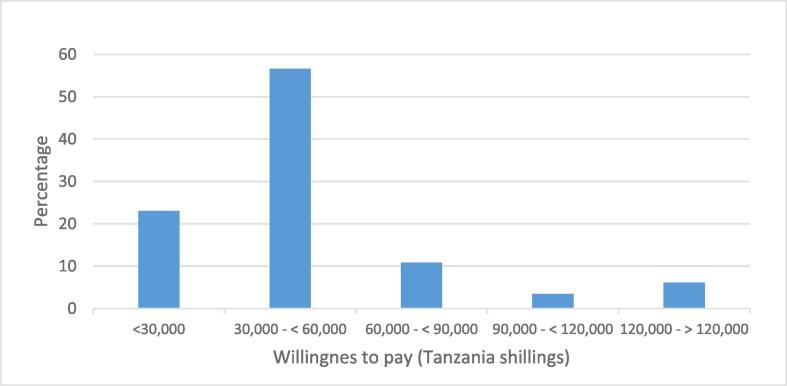
Distribution of willingness to pay for augmented iCHF.

Regarding preference for the voluntary community insurance, the result shows that more than half of the respondents preferred to pay amount equivalent to the current iCHF premium (Fig. 3).

Patients with a secondary school education or higher demonstrated a higher WTP for the current iCHF program (75.6 %), and iCHF with an additional drug package (86.9 %). Urban facility patients were significantly more inclined to pay for medication (60.8 %), the current iCHF program (76.2 %), and iCHF with an additional drug package (83.7 %) compared to rural facility patients. Retired patients displayed a higher WTP for monthly medication (61.7 %), while self-employed patients were more inclined towards iCHF with additional medication coverage (87.0 %). Patients with a positive NCD screening showed higher WTP for medication (68.1 %), the current iCHF program (63.5 %), and iCHF with additional medication coverage (80.1 %). Additionally, patients seeking care in private facilities were more disposed towards payment for medication (62.6 %), the current iCHF program (84.2 %), and iCHF with additional medication coverage (91.0 %) (Table 1). These findings underscore the influence of demographic and socioeconomic factors on patients' WTP for health services, underscoring the necessity to consider these factors in health financing scheme design and implementation.

### 4.2 Regression results

Table 2 presents the results of ordered logistic regression of WTP for paying for medication at public health facility pharmacies. After controlling for all demographic and socioeconomic characteristics, the analysis indicated certain trends, although not all results were statistically significant.

**Table 2:** Ordered logistic regression of WTP for medication.

	COR	P-value	AOR (CI)	P-value
**Gender**				
Female	1.00	−	1.00	−
Male	1.02 (0.82–1.27)	0.836	1.17 (0.94 – 1.46)	0.141
**Age (Years)**	1.01 (0.99–1.01)	0.764	0.99 (0.99–1.01)	0.256
**Education**				
No education	1.00	−	1.00	−
Primary completed	1.11 (0.86–1.43)	0.393	1.13 (0.87–1.47)	0.351
Secondary and above	1.17 (0.64–2.12)	0.603	1.33 (0.76–2.33)	0.307
**Marital status**				
Never married	1.00	−	1.00	−
Married	1.25 (0.88–1.77)	0.200	1.24 (1.01–1.52)	0.035
Separated	1.49 (1.37–1.64)	<0.001	1.63 (1.24–2.13)	<0.001
**Locality**				
Urban	1.00	−	1.00	−
Rural	0.51 (0.30–0.85)	0.010	0.53 (0.28–1.03)	0.062
**Occupation**				
Employed	1.00	−	1.00	−
Farmer	1.17 (0.87–1.58)	0.271	1.28 (0.75–2.19)	0.355
Self-employed	1.37 (1.13–1.65)	0.001	1.35 (0.75–2.45)	0.311
Retired	1.53 (0.90–2.61)	0.113	1.27 (0.75–2.14)	0.363
**Health condition reported**				
Not NCDs	1.00	−	1.00	−
NCDs	1.44 (1.18–1.77)	<0.001	1.52 (1.15 – 2.01)	0.003
**Social protection**				
OOP	1.00	−	1.00	−
iCHF	0.83 (0.66–1.03)	0.098	0.85 (0.77–0.93)	0.001
Private insurance	0.46 (0.26–0.81)	0.006	0.54 (0.33–0.88)	0.015
Exemption	0.47 (0.41–0.55)	<0.001	0.41 (0.27–0.62)	<0.001
**Facility ownership**				
Private	1.00	−	1.00	−
Public	0.62 (0.52–0.75)	<0.001	0.72 (0.59–0.62)	0.002

(COR) crude odds ratio; (AOR) adjusted odds ratio; (CI) confidence interval.

Males, patients with primary and secondary education or higher, and those who were not currently formally employed demonstrated a higher WTP for medication, but the results were not statistically significant. On the other hand, patients accessing services in rural settings demonstrated a lower WTP for medication, although this result did not reach statistical significance. Married and separated patients were 1.24 and 1.63 times respectively, more likely to pay higher for medication, and result were statistically significant. Patients currently enrolled in social health protection schemes displayed less WTP for medication, as enrollment in iCHF were 15 %, private insurance 46 %, and user fee exemption 59 % less likely, and these results were statistically significant. Patients who screened positive for non-communicable diseases (NCDs) demonstrated a higher WTP for medication with AOR (95 % CI): 1.52 (1.15 – 2.01). Furthermore, patients accessing services at public facilities displayed less WTP for medication with AOR (95 % CI): 0.72 (0.59–0.62) (Table 2).

Table 3 presents the results of ordered logistic regression analysis examining the factors associated with WTP for iCHF premiums at different bid levels. Patients with at least a secondary education were found to be more likely WTP for a higher premiums, AOR (95 % CI): 1.74 (1.25–2.39). Married (AOR (95 % CI): 2.17 (1.53–3.07)) and separated (AOR (95 % CI): 1.44 (1.16–1.79)) groups were found more likely WTP for higher premiums respectively compared to not ever married group. Conversely, patients seeking care in rural settings (AOR (95 % CI): 0.81 (034–1.88)) displayed less WTP for a higher premiums thought result was not statistically significant. Patients who have screened positive for NCDs (AOR (95 % CI): 0.89 (0.81–0.99)) demonstrated a less WTP for the higher iCHF premiums. Patients enrolled with iCHF (AOR (95 % CI): 1.09 (0.91–1.32)) reported a higher WTP for iCHF thought the result was not statistically significant. Patients with a private insurance (AOR (95 % CI): 0.06 (0.04–0.11)) and those entitled to a user fee waiver (AOR (95 % CI): 0.42 (0.24–0.72)) were willing to choose the lowest premium. Lastly, patients visiting public health facilities (AOR (95 % CI): 0.35 (0.23–0.53)) displayed less WTP for any premium (Table 3).

**Table 3:** Ordered logistic regression of WTP for augmented iCHF.

Variable	COR	P-value	AOR (CI)	P-value
**Gender**				
Female	1.00	−	1.00	−
Male	1.08 (0.64–1.82)	0.757	1.08 (0.92–1.27)	0.322
Age (Years)	0.98 (0.97–0.98)	<0.001	0.99 (0.99–1.01)	0.713
**Education**				
No education	1.00	−	1.00	−
Primary completed	1.56 (1.11–2.19)	0.009	1.20 (0.95–1.52)	0.131
Secondary and above	2.14 (1.59–2.88)	<0.001	1.74 (1.25–2.39)	0.001
**Marital status**				
Never married	1.00	−	1.00	−
Married	1.56 (1.27–1.92)	<0.001	2.17 (1.53–3.07)	<0.001
Separated	0.77 (0.71–0.84)	<0.001	1.44 (1.16–1.79)	0.001
**Locality**				
Urban	1.00	−	1.00	−
Rural	0.74 (0.40–1.36)	0.339	0.81 (034–1.88)	0.621
**Occupation**				
Employed	1.00	−	1.00	−
Farmer	0.83 (0.39–1.77)	0.636	1.09 (0.61–1.93)	0.755
Self-employed	1.39 (0.51–3.74)	0.510	1.61 (0.91–2.85)	0.105
Retired	0.49 (0.27–0.89)	0.021	1.09 (0.56–2.11)	0.791
**Health condition reported**				
Not NCDs	1.00	−	1.00	−
NCDs	0.72 (0.68–0.76)	<0.001	0.89 (0.81–0.99)	0.031
**Social protection**				
OOP	1.00	−	1.00	−
iCHF	0.75 (0.54–1.02)	0.074	1.09 (0.91–1.32)	0.331
Private insurance	0.04 (0.02–0.07)	<0.001	0.06 (0.04–0.11)	<0.001
Exemption	0.29 (0.19–0.45)	<0.001	0.42 (0.24–0.72)	0.002
**Facility ownership**				
Private	1.00	−	1.00	−
Public	0.36 (0.27–0.47)	<0.001	0.35 (0.23–0.53)	<0.001

(COR) crude odds ratio; (AOR) adjusted odds ratio; (CI) confidence interval.

## 5 Discussion

This study employed contingent valuation method to explore patient preferences to pay for iCHF and for a health insurance that includes an additional benefit package. Our findings reveal that patients demonstrate diverse preferences, even after considering socio-demographic characteristics. By utilizing client exit data, we achieved a high response rate and obtained valuable insights from individuals who have direct experience in seeking and paying for healthcare services. This approach on one hand enhances the accuracy of assessing WTP preferences, thereby improving the content validity of our study. On the other hand, it only focuses on those who managed to have access to health services.

The current study offers evidence supporting the hypothesis that access to medicines is a significant concern for patients. Our results indicate that a majority of patients exhibited a WTP for out of pocket for medications, and expressed an interest for a higher health insurance premium to enhance the availability of medicines. These findings align with those of prior studies that has underscored the challenges faced by healthcare systems in ensuring the availability of medication and supplies, as shortages often pose barriers to accessing essential healthcare services [51], [55], [56]. As expected, and consistently with the results of other studies, patients with NCDs were willing to pay more for medications [57], [58]. Additionally, our study confirms the trend observed in earlier studies that shortages of medications significantly impact patients' utilization of healthcare [59], [60].

The findings of this study emphasize the significant influence of education on patient preferences regarding payment for iCHF premiums with additional medicine packages. Patients with a secondary education or higher were more likely to demonstrate their WTP for insurance premiums. This may be attributed to their greater capability to appreciate the benefits of having a health insurance against the unforeseen medical expenses [61], [62]. Furthermore, previous studies have similarly highlighted that higher educational levels correlate with increased health literacy, which in turn enhances the understanding and valuation of health insurance products [25], [36]. These insights underscore the importance of educational interventions to improve health insurance uptake and financial preparedness among populations with lower educational attainment.

Furthermore, patients residing in urban areas exhibited a greater inclination to pay for medication, potentially due to their higher income and increased access to economic opportunities, although this association did not reach statistical significance [63], [64]. On the contrary, patients enrolled in insurance schemes or benefiting from user fee waivers were less likely to pay out of pocket for medication, as they expected to access services without incurring additional costs. Lastly, patients seeking care at private facilities displayed a higher WTP for medicines, highlighting the discrepancy between the current designs of voluntary schemes, which primarily cater to care received in public facilities. Previous studies have indicated that private healthcare facilities are often perceived to provide higher quality care due to better management and services, which drives patients' WTP more for these perceived benefits [65]. Additionally, this supports findings that patients are willing to pay premiums for improved quality and services in private settings, aligning with the concept of increased WTP for voluntary health schemes that offer access to more benefits [66].

This study contributes to the growing body of evidence that interventions aimed at improving voluntary health insurance schemes should be tailored to meet the specific needs and preferences of different patient groups. Additionally these schemes should offer various premiums levels and include comprehensive medication package benefits. While previous research has generally shown evidence to support scaling up for voluntary health insurances (1, 8, 14, 42), our study found that the scale-up of iCHF as one of the scheme to achieve health insurance for all, may have varying impacts on patient preferences based on socio-demographic factors [5], [14], [29], [67]. This underscores the importance of targeted interventions that consider the specific needs and preferences of different patient subgroups.

Our findings are consistent with previous studies that have highlighted the need for multiple strategies to address socio-economic disparities in access to health insurance [7], [15], [17]. However, it is important to note that policy decisions based solely on the opinions of better off citizens may lead to low reenrollment rates and affordability issues for intended beneficiaries. Therefore, it is critical to consider the perspectives and needs of all population groups when developing and implementing voluntary insurance schemes for all, such as iCHF [31], [68], [69].

The results of this study may have policy implications for the voluntary iCHF program in Tanzania. The strong preference for the current iCHF premium, despite low actual enrollment, suggest limited potential for increasing premiums from the current voluntary insurance base, which covers the majority of individuals. These findings highlight the need for continued government efforts to facilitate easy access to affordable and well-functioning health insurance. Furthermore, group differences should be taken into consideration, as patient screened positive with NCDs and accessing care in public health facilities showed unwillingness to pay for augmented iCHF. Previous studies have indicated that decision to enroll or dropout for patient with chronic condition associated with perception of quality of services, insurance management and income status [36], [69].

### 5.1 Limitation and strength

The study is among the first to investigate voluntary insurance schemes in Tanzania, and the results provide valuable insights into the preferences of healthcare users in this setting. However, there are limitations to the study that should be considered when interpreting the results. One limitation is that the study sampled only healthcare users, and the results may not be generalizable to non-healthcare users. Additionally, the contingent valuation methodology used a restricted set of bids that used to elicit WTP that may affect the realism of the study. Furthermore, respondents' trust in bids that are currently not available on the insurance market may have affected their preferences. Another significant limitation is the potential for endogeneity, which could arise from unobserved factors influencing both the respondents' WTP for insurance and their healthcare utilization patterns. Additionally, the lack of a counterfactual limits the ability to establish a causal relationship between the intervention and observed outcomes. Furthermore, the study used the consumer choice model, and it is likely that health promotion activities affects choices of health insurance as the intervention goes with public health promotions [70], and the technological developments [71]. Nonetheless, these limitations do not undermine the relevance of the study's findings that provide valuable insights into the preferences of patients regarding voluntary insurance schemes for healthcare in Tanzania. Future studies could explore the preferences of non-healthcare users and examine the impact of alternative interventions and policies on healthcare access and utilization. Additionally, studies could examine the role of technology and mobile platforms in increasing enrollment and improving healthcare access among underserved populations.

### 5.2 Policy implications

This study underscores critical considerations for stakeholders involved in designing and implementing health insurance schemes in low-income settings. Our findings reveal nuanced patient preferences regarding premium costs and benefit packages, indicating that affordability and service quality are pivotal factors influencing enrollment decisions. Policymakers should prioritize affordability by carefully assessing premium costs to ensure they align with patients' financial capacities, thereby enhancing enrollment rates and sustainability of the insurance scheme. Furthermore, the government's role in addressing healthcare sector deficiencies is crucial; efforts should focus on strengthening collaboration between insurance providers and healthcare facilities to improve service delivery and ensure the availability of high-quality care.

Offering additional benefit packages that cater to diverse patient needs, such as comprehensive medication coverage, could enhance the attractiveness of voluntary health insurance schemes. This approach aligns with our findings that highlight the demand for expanded benefits, particularly among patients with chronic conditions and those accessing care in public health facilities. Moreover, recognizing the socio-economic disparities in access to healthcare, policymakers should tailor interventions to address the specific needs of different patient groups, ensuring equitable access to insurance benefits.

Importantly, the study suggests that policy decisions should not solely rely on the preferences of more affluent citizens but should also consider the perspectives of all population segments to foster inclusivity and enhance the scheme's effectiveness. By incorporating these insights into policy design, Tanzania can potentially overcome barriers to health insurance enrollment. These recommendations underscore the importance of evidence-based policymaking that responds to the diverse preferences and needs of healthcare consumers, ultimately contributing to the long-term sustainability and success of voluntary health insurance initiatives in Tanzania and similar contexts.

## Funding

This manuscript is an output from the project: Health systems governance for an inclusive and sustainable social health protection in Ghana and Tanzania funded by the Swiss Programme for Research on Global Issues for Development programme, phase two January 2018 – December 2022. The project involved a consortium of 3 partners: Swiss Tropical and Public Health Institute, Ifakara Health Institute Tanzania and University of Ghana. The funders had no role in study design, data collection and analysis, decision to publish, or preparation of the manuscript.

## CRediT authorship contribution statement

**Kassimu Tani:** Writing – review & editing, Writing – original draft, Methodology, Formal analysis, Data curation, Conceptualization. **Brianna Osetinsky:** Writing – review & editing, Validation, Data curation, Conceptualization. **Sally Mtenga:** Writing – review & editing, Validation, Supervision, Data curation. **Günther Fink:** Writing – review & editing, Validation, Supervision. **Fabrizio Tediosi:** Writing – review & editing, Writing – original draft, Validation, Supervision, Funding acquisition, Conceptualization.

## Declaration of competing interest

The authors declare that they have no known competing financial interests or personal relationships that could have appeared to influence the work reported in this paper.
