# Supplementary material for: Patient’s willingness to pay for improved community health insurance in Tanzania
Source: Health Policy Open. 2024 Sep 29;7:100130. doi: 10.1016/j.hpopen.2024.100130 (PMC11497436; doi:10.1016/j.hpopen.2024.100130)
Supplement: Supplementary Data 1 [file mmc1.docx]

**Supplementary Files:**

S1 Table: Supplementary material

S2 Table: Starting and ending point for the bidding game iteration for to estimate the respondent willingness to pay for the medication and iCHF premium with additional medication coverage

S3 Table: Description of dependent and independent variables

S4 Table: The Brant test for the proportional odds assumptions

S5 Table: Linktest results for regression results in Table (2)

S6 Table: Linktest results for regression results in Table (3) S1 Figure: Willingness to pay and quantity of improved insurance coverage

S2 Figure: Conceptual framework (authors design)

S3 Figure: Map of Tanzania showing study districts: Same and Kilombero

S4 Figure: Statistical summary of the responses to double bounded dichotomous choice question on medication bids

S5 Figure: Statistical summary of the responses to double bounded dichotomous choice question on iCHF premium with additional medication package

**Supplementary materials**

**S1 Table: Supplementary material**

| Attributes | Questionnaire prompt | Level |
| --- | --- | --- |
| Monthly medication expenditure | “One of the hard things with chronic conditions is that you need to continually take medicine for a long time, instead of a treatment that is done when your medication is finished. Now imagine you need medicine to treat a chronic disease, and that you would need to buy the medicine every month. In other words at the end of one month, you would need to purchase enough medicine to last you until the end of the next month when you would return again to buy more medicine, and you would need to do this all year long. I am going to ask you about how much you would be willing to pay for those medications out of pocket. If you have insurance this does not include the cost of the premium, but would be an extra cost that you might incur if the medicine is not paid for by your insurance, or if the medicine is not available at a pharmacy that is covered by your insurance.”  **The questions:**  “Would you be willing to pay at least 5,000 TSH every month for medications, which is a total of 60,000TSH throughout the year?”  “Would you be willing to pay at least 2,500 TSH every month for medications, which is a total of 30,000TSH throughout the year?”  “Would you be willing to pay at least 1,000 TSH every month for medications, which is a total of 12,000TSH throughout the year?”  “Would you be willing to pay at least 10,000 TSH every month for medications, which is a total of 120,000TSH throughout the year?”  “What is the most you would be willing to pay for your medication every month? If you would not be willing to pay any money out of pocket please say 0.” | 1,000, 2,500, 5,000 and 10,000 Tanzanian shillings |
| Current iCHF premium | “iCHF is a community health insurance fund that costs 30,000TSH per year for a family of up to six people. For this price you can access care at public health facilities and should not have to pay additional shillings for registration, consultation, or tests. If you are prescribed medication you should not have to pay if you receive the medication in the public pharmacy, but you will have to pay for the medication if you get it from a private pharmacy. However, you might be referred to a different facility like the hospital or be asked to return on a different day for chronic disease care.”  **The question:**  “Would you would be willing to pay the 30,000 TSH for this program for your family?” | Yes  and  No |
| ICHF premium with medication to private pharmacy | “Now consider another scenario, where the insurance fund also includes costs of prescribed medication at any private pharmacy if the public pharmacy has a stock out. This is in addition to the existing access to care at public health facilities, so you would still not have to pay additional shillings for registration, consultation, or tests, OR medications. However, you might be referred to a different facility like the hospital or be asked to return on a different day for chronic disease care.”  **The questions:**  “If the annual costs was 60,000TSH per year for a family of up to six people would you be willing to pay for this insurance?”  “If the annual costs was 30,000TSH per year for a family of up to six people would you be willing to pay for this insurance?”  “If the annual costs was 90,000TSH per year for a family of up to six people would you be willing to pay for this insurance?”  “If the annual costs was 120,000TSH per year for a family of up to six people would you be willing to pay for this insurance?”  “How much is the most you would be willing to pay per year for a family of six for this insurance?” | 30,000, 60,000, 90,000 and 120,000 Tanzanian shillings |

***S2 Table: Starting and ending point for the bidding game iteration for to estimate the respondent willingness to pay for the medication and iCHF premium with additional medication coverage***

|  | **Medication - Tshs** | **iCHF premium with medication - Tshs** |
| --- | --- | --- |
|  | 1,000 | 30,000 |
|  | 2,500 | 60,000 |
| Starting point | 5,000 | 90,000 |
|  | 10,000 | 120,000 |

**S3 Table: Description of dependent and independent variables**

| Variable | Measurement |
| --- | --- |
| Willingness to pay for medication | <1000; 1000 - <2500; 2500 - <5000; 5000 - <10000; and >10000 Tanzania shilling |
| Willingness to pay for augmented iCHF | <30000; 30000 - <60000; 60000 - <90000; 90000 - <120000; and >120000 Tanzania shilling |
| Gender | 0= Female 1= Male |
| Age of Respondents | 0= Below 35 1= 35 – 55 3= Above 55 |
| Education level | 0= No education, 1= Completed primary, 3=Secondary and above |
| Marital status | 0= never married 1=Married 3=Separated |
| Locality | 0= Urban, 1 = Rural |
| Occupation | 0= Employed, 1= Farmer, 2= Self-employed, 3= Retired |
| Health condition reported | 0= Not having non communicable diseases, 1=Have non-communicable condition |
| Social protection | 0= Out of pocket, 1= improved Community health fund, 2=National health insurance fund, 3=Private insurance, 4 Exemption |
| District | 0= Kilombero, 1=Same |
| Facility ownership | 0=Private 1= Public |
| Facility level | 0=Dispensary, 1= Health centre 3. Hospital |

**S4a Table: The Brant test for the proportional odds assumptions for WTP for medication**

| **Variable** | **Chi-square** | **p-value** | **Degrees of Freedom** |
| --- | --- | --- | --- |
| All | 78.12 | 0.16 | 27 |
| Gender | 8.59 | 0.13 | 3 |
| Age category | 0.93 | 0.81 | 3 |
| Education level | 21.16 | 0.26 | 3 |
| Marital status | 4.63 | 0.20 | 3 |
| Locality | 8.11 | 0.09 | 3 |
| Occupation | 4.01 | 0.26 | 3 |
| Health condition reported | 1.43 | 0.69 | 3 |
| Social protection | 6.55 | 0.08 | 3 |
| Facility level | 0.39 | 0.94 | 3 |

**S4b Table: The Brant test for the proportional odds assumptions WTP for augmented iCHF**

| **Variable** | **Chi-square** | **p-value** | **Degrees of Freedom** |
| --- | --- | --- | --- |
| All | 43.67 | 0.20 | 27 |
| Gender | 3.57 | 0.31 | 3 |
| Age category | 3.83 | 0.28 | 3 |
| Education level | 4.21 | 0.24 | 3 |
| Marital status | 3.92 | 0.27 | 3 |
| Locality | 4.55 | 0.21 | 3 |
| Occupation | 10.51 | 0.11 | 3 |
| Health condition reported | 3.60 | 0.31 | 3 |
| Social protection | 3.45 | 0.33 | 3 |
| Facility level | 1.75 | 0.63 | 3 |

**S5 Table: Linktest results for regression results in Table (2)**

| Variables  Coefficient (Confidence interval) |  | |
| --- | --- | --- |
|  | Coef (95% CI) | p-value |
| _hat | 1.046(0.83-1.25) | 0.000 |
| _hatsq | 0.135(-0.15-0.42) | 0.361 |
| Number of observations | 1737 | |
| LR Chi^2^ | 117.910 | |
| Prob > chi^2^ | 0.000 | |
| Pseudo R2 | 0.023 | |

**S6 Table: Linktest results for regression results in Table (3)**

| Variables  Coefficient (Confidence interval) |  | |
| --- | --- | --- |
|  | Coef (95% CI) | p-value |
| _hat | 1.001(0.84-1.17) | 0.000 |
| _hatsq | -0.013(-0.12-0.94) | 0.814 |
| Number of observations | 1310 | |
| LR Chi^2^ | 192.26 | |
| Prob > chi^2^ | 0.000 | |
| Pseudo R2 | 0.062 | |

***S1 Figure: Willingness to pay and quantity of improved insurance coverage***

WTP (Tshs)

|

| _____________________

| /

| /

| /

| /

|_______/

|

|______________________________ Quantity of Improved Insurance

This curve visually shows that as the quantity of improved insurance coverage increases, willingness to pay increases initially at a decreasing rate until it levels off.

***S2 Figure: Conceptual framework (author’s design)***


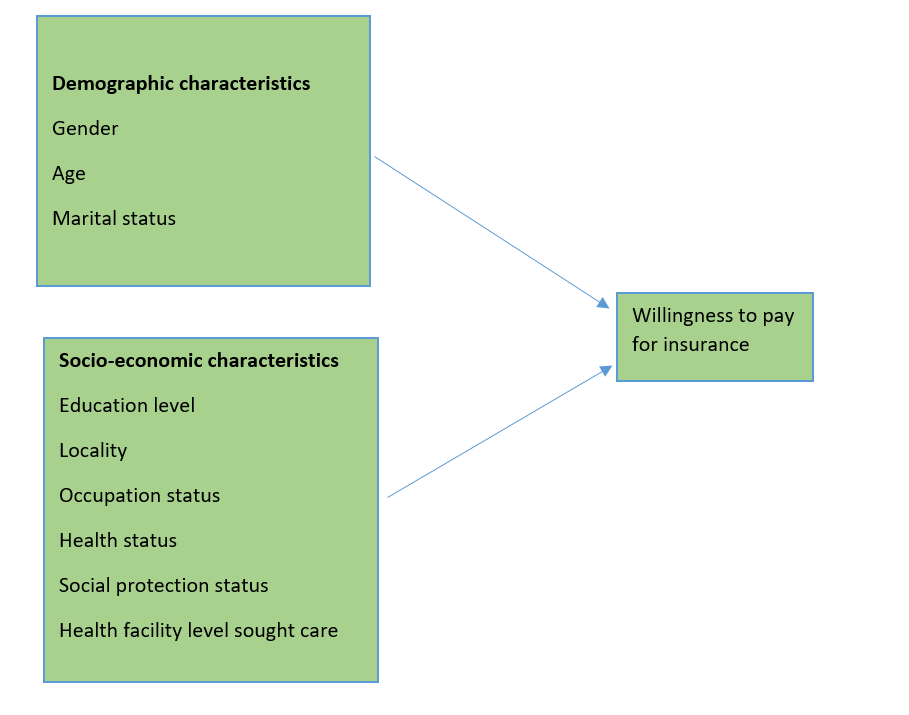


***S3 Figure: Map of Tanzania showing study districts: Same and Kilombero***


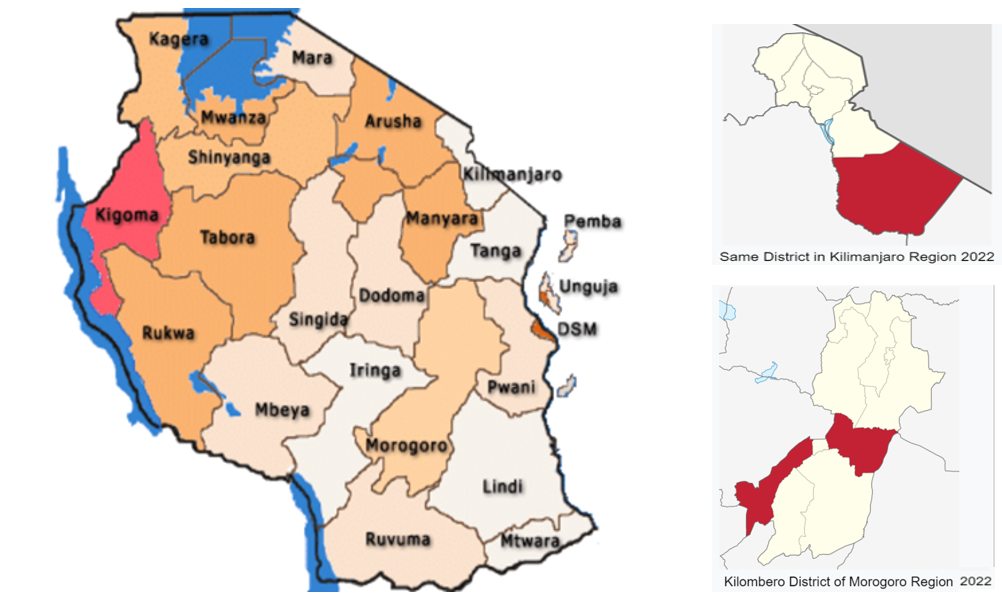


***S4 Figure: Statistical summary of the responses to double bounded dichotomous choice question on medication bids***

***S5 Figure:*** ***Statistical summary of the responses to double bounded dichotomous choice question on iCHF premium with additional medication package***
